# Supplementary material for: Tunable metal-insulator transition, Rashba effect and Weyl Fermions in a relativistic charge-ordered ferroelectric oxide
Source: Nat Commun. 2018 Feb 5;9:492. doi: 10.1038/s41467-017-02814-4 (PMC5799170; doi:10.1038/s41467-017-02814-4)
Supplement: Supplementary file 1 — Supplementary Information [file 41467_2017_2814_MOESM1_ESM.pdf]

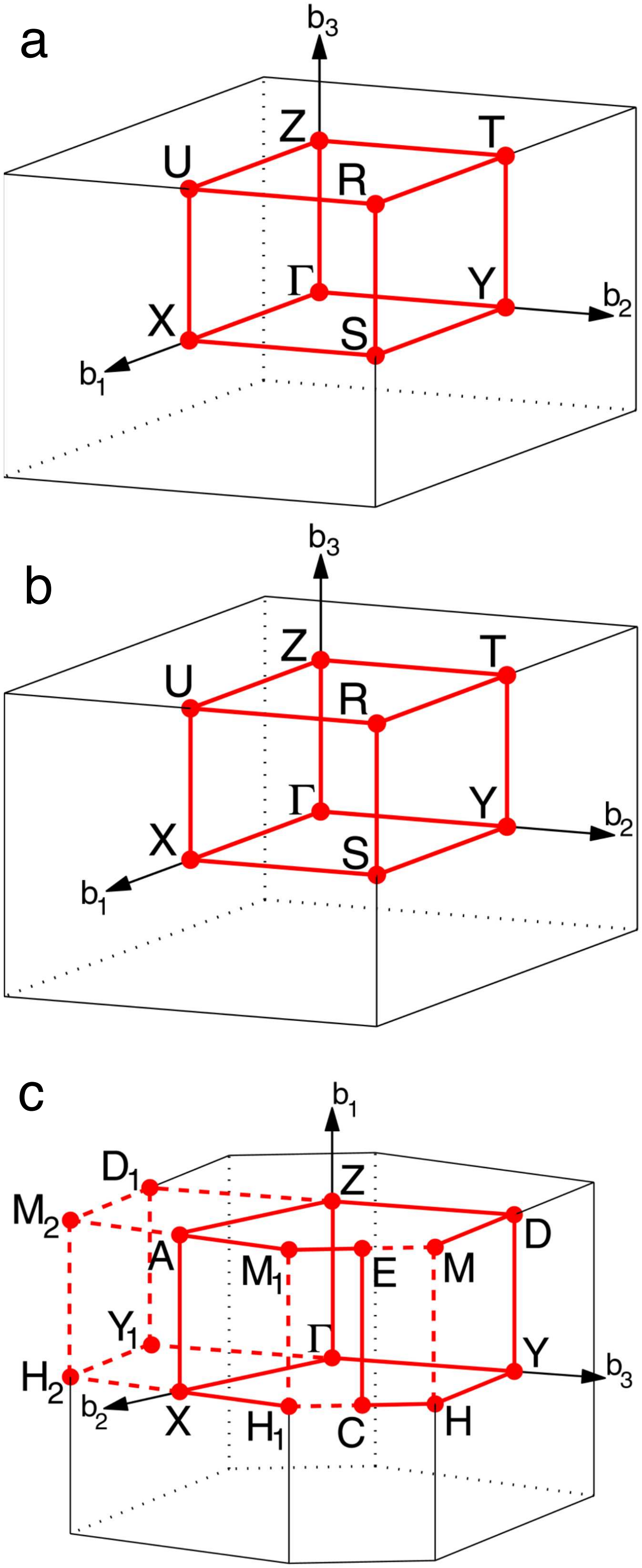

Supplementary Figure 1: Brillouin zone of Ag_2BiO_3 in $Pnna$ (a), $Pnn2$ (b), and Pn (c) phases.

Supplementary Table 1: Lattice parameters, Bi-O bond length (Å), band gap (E_g, eV) of the fully relaxed crystal structures by using LDA, PBE, PBEsol, and SCAN and experimental crystal structures of *Pnn*2 and *Pn* phases. Energy differences (ΔE, meV/atom) and polar structure distortion amplitudes (Q, Å) of *Pnn*2 with respect to *Pnna* (Γ_2^-) and *Pn* with respect to *Pnn*2 (Γ_4) computed using these functionals also are indicated.

	a (Å)	b (Å)	c (Å)	beta (°)	Volume	Bi^{3+}-O	Bi^{3+}-O	Bi^{3+}-O	Bi^{5+}-O	Bi^{5+}-O	Bi^{5+}-O	E_g	ΔE	Q
							*Pnn*2 (No. 34)							
LDA	5.95623	6.23420	9.38495		348.49	2.3255	2.3121	2.2881	2.1845	2.1636	2.1845	0.65	-7.52	0.6709
PBE	6.16207	6.31588	9.75448		379.63	2.3667	2.2422	2.2258	2.2422	2.2258	2.1453	0.43	-4.72	0.4179
PBEsol	6.04299	6.24922	9.53532		360.09	2.3417	2.3310	2.2959	2.2080	2.1894	2.1161	0.53	-5.34	0.5323
SCAN	6.03989	6.29047	9.58744		364.26	2.3452	2.3455	2.3236	2.1968	2.1901	2.1036	0.84	-11.22	0.5165
Exp. [5]	5.9830	6.3239	9.5762		362.33	2.3557	2.3355	2.3195	2.1635	2.1316	2.0818	0.7 [6]		0.5770
							Pn (No. 7)							
LDA	9.36397	6.23743	5.95012	90.42	347.52	2.3275	2.3194	2.2905	2.1863	2.1627	2.0951	0.66	0.00	0.0819
						2.3234	2.3073	2.2929	2.1834	2.1614	2.0924			
PBE	9.76345	6.31598	6.16146	90.32	379.95	2.3688	2.3646	2.3334	2.2455	2.2270	2.1470	0.54	0.00	0.1273
						2.3651	2.3499	2.3284	2.2406	2.2259	2.1453			
PBEsol	9.54891	6.24390	6.04036	90.58	360.14	2.3416	2.3390	2.3001	2.2105	2.1906	2.1180	0.53	0.00	0.1900
						2.3408	2.3195	2.2911	2.2053	2.1898	2.1139			
SCAN	9.58266	6.29418	6.03485	90.15	363.99	2.3455	2.3365	2.3259	2.1966	2.1883	2.0984	0.87	0.00	0.1183
						2.3438	2.3349	2.3170	2.1923	2.1848	2.0977			
Exp. [5]	9.58082	6.31001	5.95492	92.48	359.67	2.409	2.309	2.263	2.176	2.146	2.109			0.7313
						2.395	2.300	2.260	2.165	2.126	2.056			

Supplementary Table 2: Lattice constants and Wyckoff positions of fully relaxed and experimental crystal structures Pnna, Pnn2, and Pn.

		Theory in this work			Experiment from Ref[**?**]		
			Pnna (No. 52)				
a		6.04299					
b		6.24922					
c		9.53532					
Atom	Wyckoff	x	y	z	x	y	z
Ag1	4a	0.0000	0.0000	0.0000			
Ag2	4d	0.7196	0.2500	0.2500			
Bi1	4c	0.2500	0.0000	0.3959			
O1	8e	0.5662	0.6852	0.0640			
O2	4d	0.3566	0.2500	0.2500			
			*Pnn*2 (No. 34)				
a		6.04036			5.9830		
b		6.24390			6.3239		
c		9.54891			9.5762		
Atom	Wyckoff	x	y	z	x	y	z
Ag1	4c	0.2538	-0.0004	0.4966	0.2528	-0.0016	0.4954
Ag2	4c	0.0286	0.2409	0.2558	0.0256	0.2512	0.2502
Bi1	2a	0.0000	0.0000	0.8930	0.0000	0.0000	0.8992
Bi2	2b	0.0000	0.5000	0.6040	0.0000	0.5000	0.6074
O1	4c	0.8855	0.2857	0.2401	0.1954	0.6777	0.4350
02	4c	0.3249	0.1868	0.0583	0.8296	0.1805	0.0540
O3	4c	0.6969	0.6832	0.4326	0.3920	0.2903	0.2459
			Pn (No. 7)				
a		6.0461			5.9549		
b		6.2488			6.3100		
c		9.5472			9.5808		
β		90.4422			92.4823		
Atom	Wyckoff	x	y	z	x	y	z
Ag11	2a	0.2539	0.2475	0.4930	0.2674	0.2336	0.4895
Ag12	2a	0.7468	0.2482	0.4974	0.7499	0.2488	0.5081
Ag21	2a	0.0261	0.4944	0.2554	0.0107	0.5148	0.2496
Ag22	2a	0.9687	0.0093	0.2538	0.9730	0.9955	0.2375
Bi1	2a	0.9985	0.2500	0.8961	0.0042	0.2537	0.9032
Bi2	2a	0.9997	0.7470	0.6068	0.0048	0.7411	0.6081
O11	2a	0.8248	0.4401	0.0561	0.8279	0.4585	0.0371
O12	2a	0.1749	0.0663	0.0604	0.1663	0.0950	0.0719
O21	2a	0.1941	0.9313	0.4329	0.1936	0.9101	0.4327
O22	2a	0.8020	0.5655	0.4316	0.8008	0.5694	0.4390
O31	2a	0.3830	0.5404	0.2372	0.3813	0.5540	0.2287
O32	2a	0.6103	0.9725	0.2455	0.6028	0.9885	0.2616

Supplementary Table 3: Positions and chirality of four pairs of Weyl nodes in the first BZ. The position (k_x, k_y, k_z) are in units of reciprocal lattices. All of them are related by crystal and time-reversal symmetries.

Weyl point	k_x	k_y	k_z	Chirality
W1	0.4975	0.4725	0.4988	+
W2	-0.4975	0.4725	0.4988	-
W3	-0.4975	-0.4725	0.4988	+
W4	0.4975	-0.4725	0.4988	-
W5	0.4975	0.4725	-0.4988	+
W6	-0.4975	0.4725	-0.4988	-
W7	-0.4975	-0.4725	-0.4988	+
W8	0.4975	-0.4725	-0.4988	-

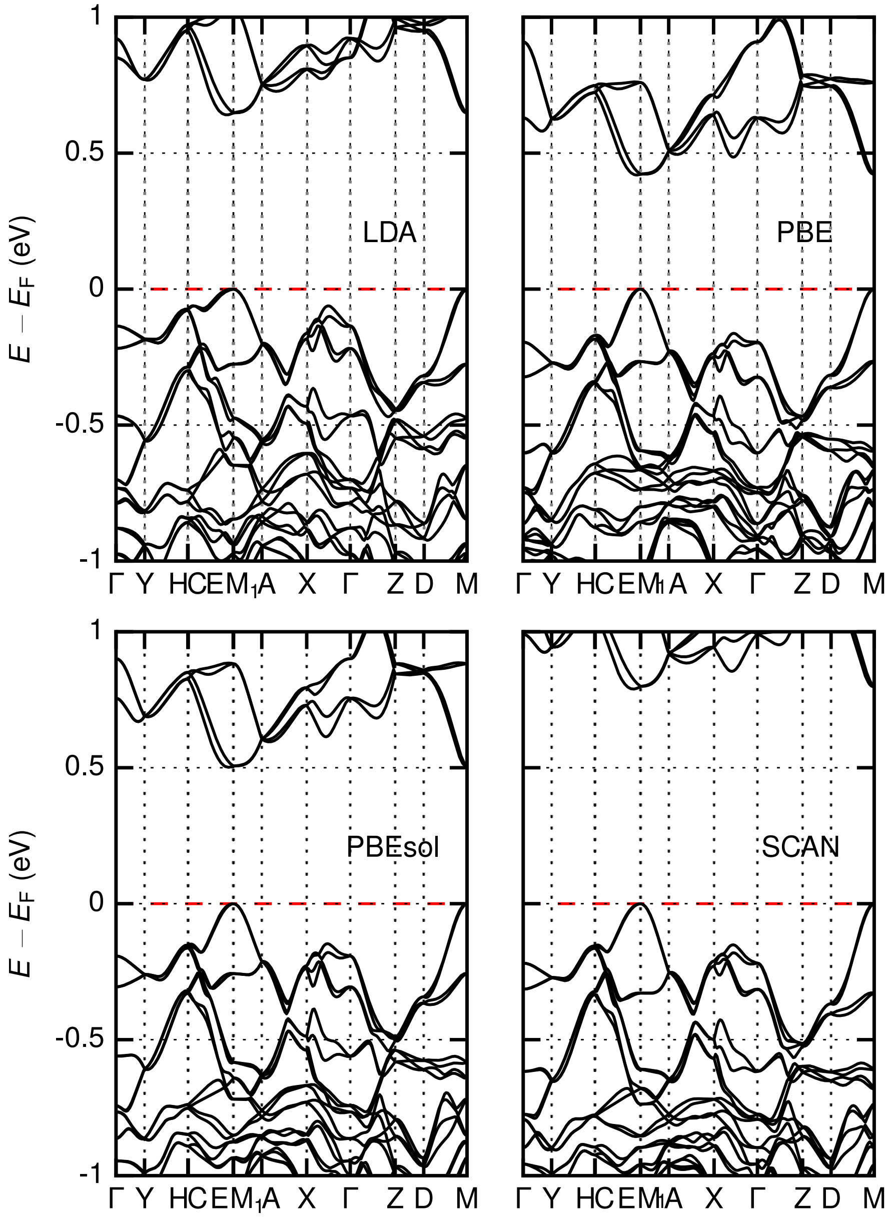

Supplementary Figure 2: Band structures of Pn phase calculated using LDA, PBE, PBEsol, and SCAN

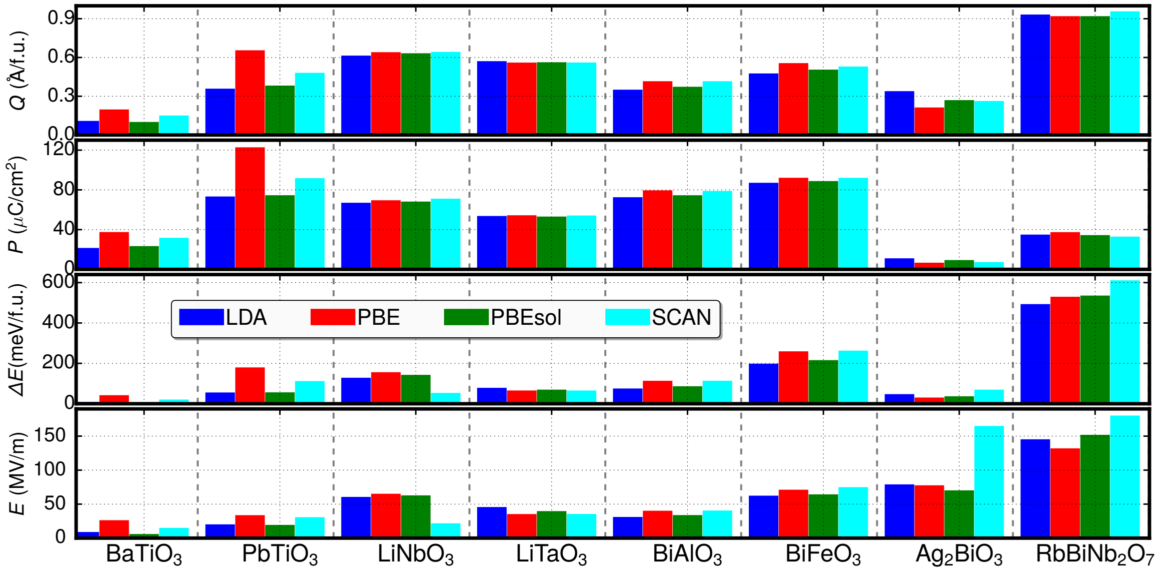

Supplementary Figure 3: The calculated polar distortion amplitude (Q), spontaneous polarization (P), energy difference between paraelectric phase and ferroelectric phase (ΔE), and the electrical field (E) required to switch from -P to +P for Ag_2BiO_3 and other well-known and switchable ferroelectric materials using different functionals.

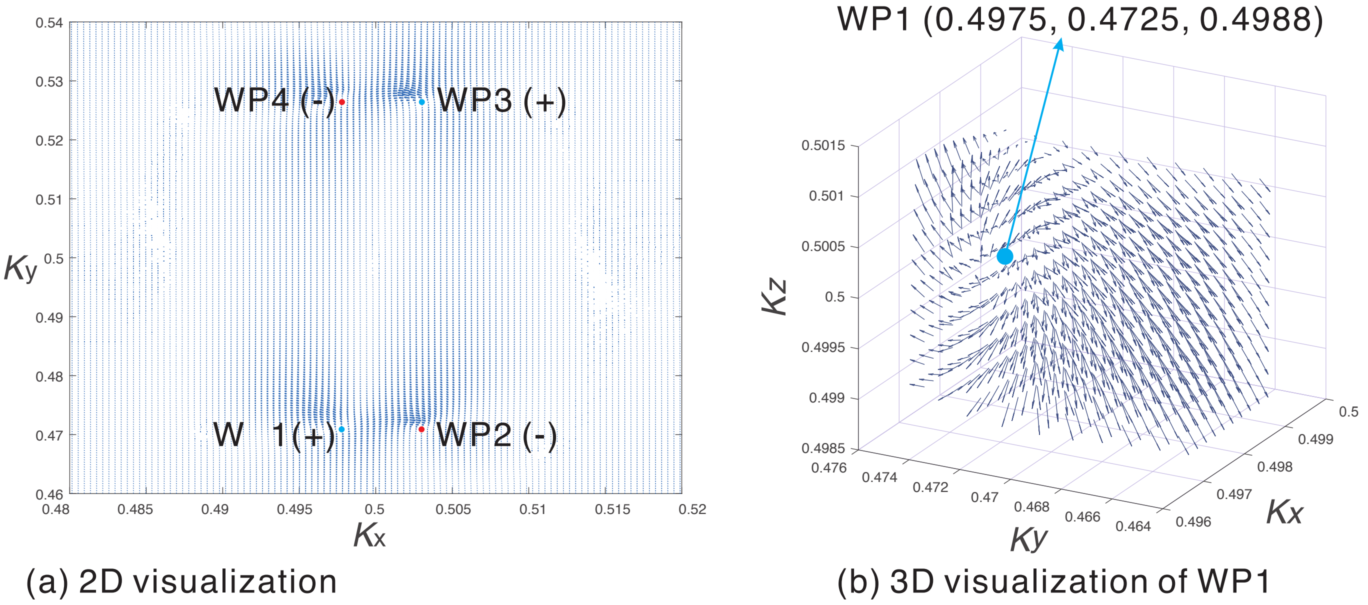

Supplementary Figure 4: (a) 2D and 3D plots of four Weyl points (WP1, WP2, WP3, and WP4). (b) 3D Berry curvature of WP1.

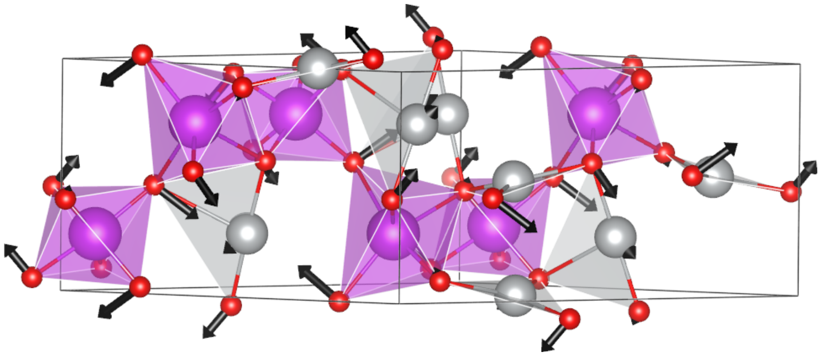

Supplementary Figure 5: Atomic displacements of the structure distortion from $Pnn2$ to Pn. The main distortions are rotation of $Bi^{5+}O_6$ octahedra and tilting of $Bi^{3+}O_6$ octahedra. The displacement amplitude is scaled to the largest displacement in this distortion.

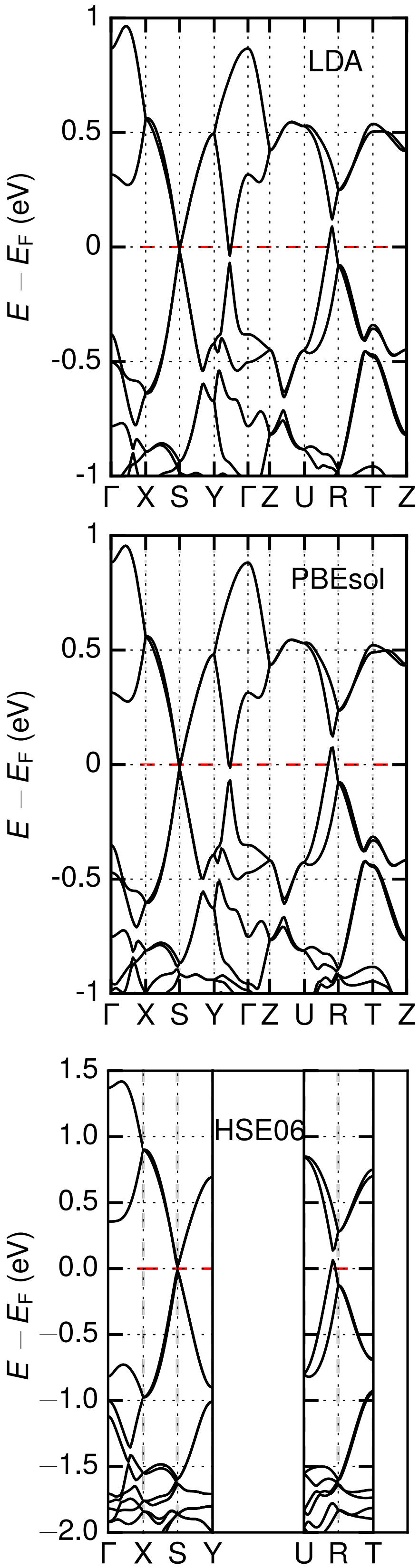

Supplementary Figure 6: Band structures of $Pnna$ phase calculated using LDA, PBE, PBEsol, SCAN, and HSE06

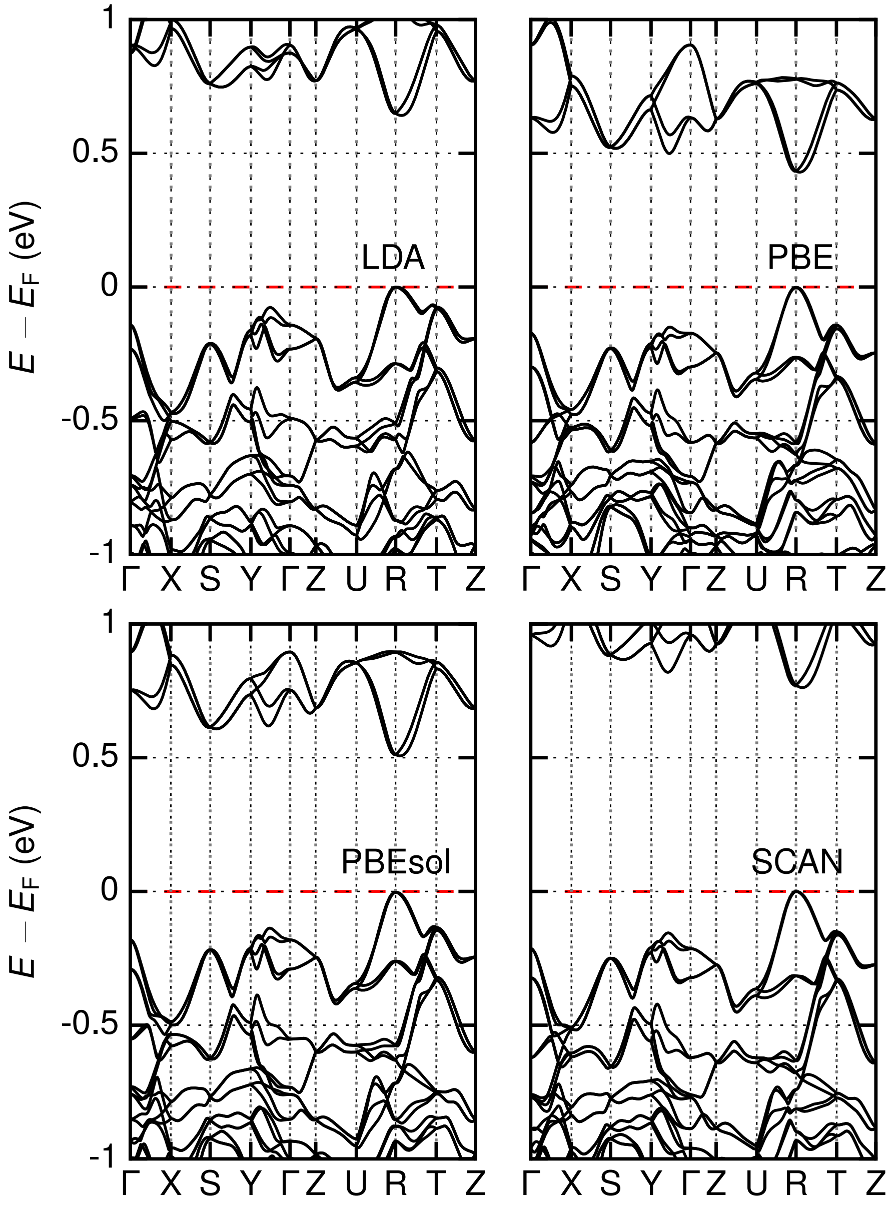

Supplementary Figure 7: Band structures of $Pnn2$ phase calculated using LDA, PBE, PBEsol, and SCAN

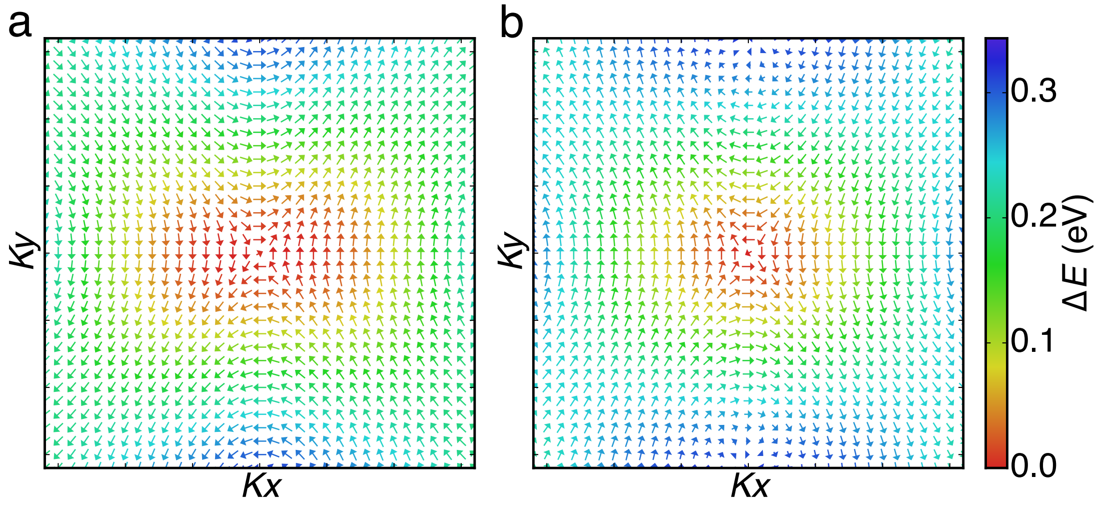

Supplementary Figure 8: The inner (a) and outer (b) bands spin texture of Ag_2BiO_3 in $Pnn2$ phases with negative polarization (-$\vec{P}$). he color-code indicates the energy level with respect to the bottom of conduction band.

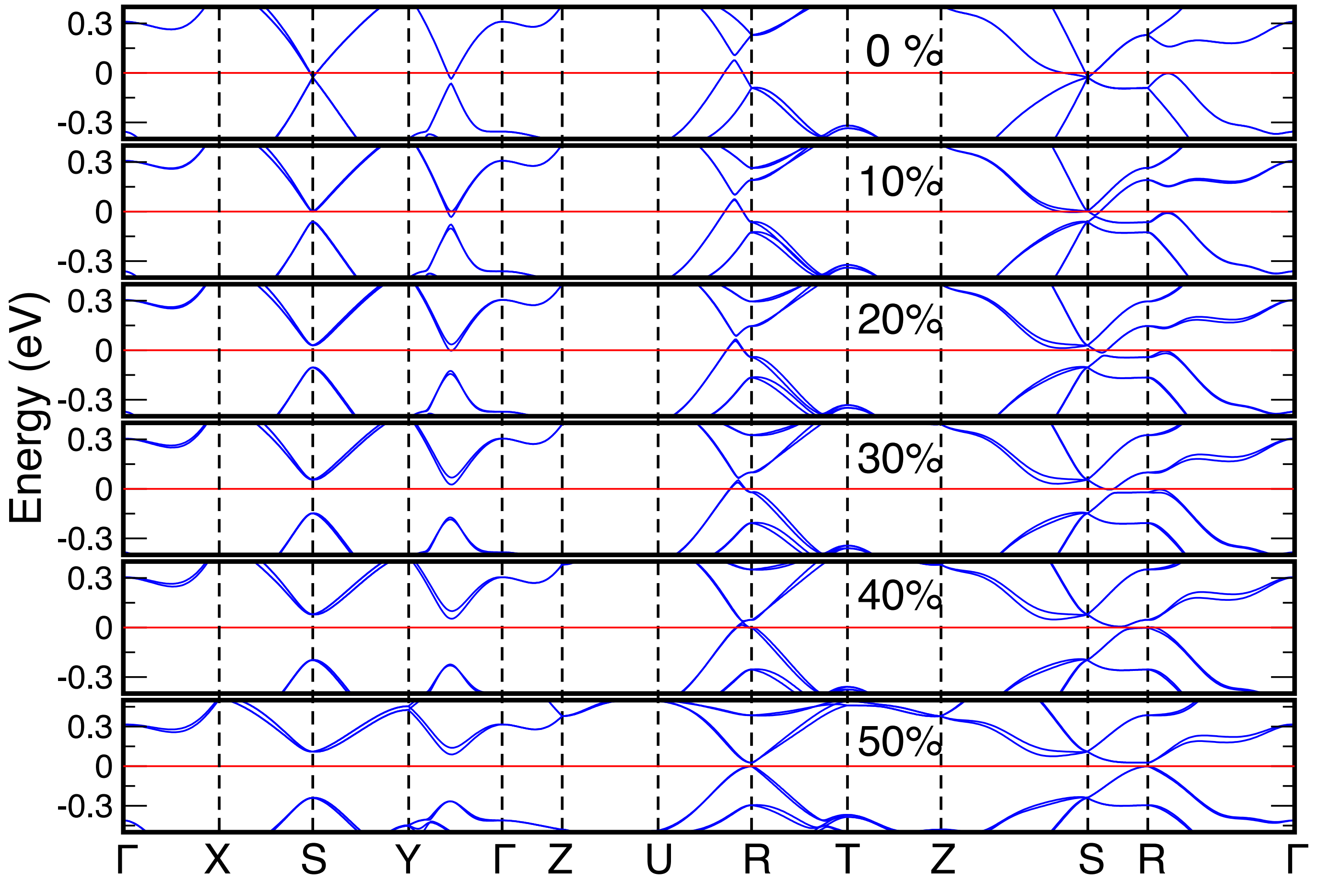

Supplementary Figure 9: Band structure evolution with amplitude of the polar Γ_2^- mode from 0% to 50%.

Supplementary References:

[1] D. M. Ceperley and B. J. Alder, Phys. Rev. Lett. **45**, 566 (1980).
[2] J. P. Perdew, K. Burke, and M. Ernzerhof, Phys. Rev. Lett. **77**, 3865 (1996)
[3] J. Sun, A. Ruzsinszky, and J.P. Perdew, Phys. Rev. Lett. **115**, 036402 (2015)
[4] Aliaksandr V. Krukau, Oleg A. Vydrov, Artur F. Izmaylov, and Gustavo E. Scuseria, J. Phys. Chem. **125**, 224106 (2006)
[5] Christian P.M. Oberndorfera, Robert E. Dinnebiera, Richard M. Ibbersonb, and Martin Jansen, "Charge ordering in Ag_2BiO_3", Solid State Sci. **8**, 267 (2006).
[6] S. Deibele and M. Jansen, "Bismuth in Ag_2BiO_3: Tetravalent or internally disproportionated?" J. Solid State Chem. **147**, 117-121 (1999).
